# Corticosterone Methyl Oxidase Deficiency Type 1 with Normokalemia in an Infant

**DOI:** 10.4274/jcrpe.2824

**Published:** 2016-09-01

**Authors:** Ala Üstyol, Mehmet Emre Atabek, Norman Taylor, Matthew Chun-wing Yeung, Angel O. K. Chan

**Affiliations:** 1 Necmettin Erbakan University Faculty of Medicine, Department of Pediatric Endocrinology and Diabetes, Konya, Turkey; 2 King’s College Hospital, Clinic of Clinical Biochemistry, London, United Kingdom; 3 The University of Hong Kong, Queen Mary Hospital, Clinic of Pathology, Pokfulam, Hong Kong

**Keywords:** Corticosterone methyl oxidase, salt wasting, CYP11B2 gene, failure to thrive

## Abstract

Isolated aldosterone synthase deficiency may result in life-threatening salt-wasting and failure to thrive. The condition involves hyperkalemia accompanying hyponatremia. Two types of aldosterone synthase deficiency may be observed depending on hormone levels: corticosterone methyl oxidase type 1 (CMO 1) and CMO 2. Herein, we describe a Turkish infant patient with aldosterone synthase deficiency who presented with failure to thrive and salt wasting but with normal potassium levels. Urinary steroid characteristics were compatible with CMO I deficiency. Diagnosis of aldosterone synthase deficiency was confirmed by mutational analysis of the CYP11B2 gene which identified the patient as homozygous for two mutations: c.788T>A (p.Ile263Asn) and c.1157T>C (p.Val386Ala). Family genetic study revealed that the mother was heterozygous for c.788T>A and homozygous for c.1157T>C and the father was heterozygous for both c.788T>A and c.1157T>C. To the best of our knowledge, this is only the second Turkish case with a confirmed molecular basis of type 1 aldosterone synthase deficiency. This case is also significant in showing that spot urinary steroid analysis can assist with the diagnosis and that hyperkalemia is not necessarily part of the disease.

**WHAT IS ALREADY KNOWN ON THIS TOPIC?**

Isolated aldosterone synthase deficiency may result in life-threatening salt-wasting and failure to thrive. To the best of our knowledge, the literature contains only one case of a Turkish patient with aldosterone synthase deficiency with confirmed mutation in the CYP11B2 gene.

**WHAT THIS STUDY ADDS?**

We described a Turkish patient with aldosterone synthase deficiency presenting with failure to thrive and salt-wasting but with normal potassium levels in infancy. Diagnosis of aldosterone synthase deficiency was confirmed by mutational analysis of the CYP11B2 gene.

## INTRODUCTION

Aldosterone is essential to life due to two important functions - sodium excretion and intravascular volume regulation. It is synthesized by aldosterone synthase and encoded by CYP11B2 gene on the long arm of chromosome 8. Beginning from 11-deoxycorticosterone in the zona glomerulosa of the adrenal cortex, aldosterone synthase catalyzes the sequential activities of 11β-hydroxylase, 18-hydroxylase, and, finally, 18-methyl oxidase, the final three steps in aldosterone synthesis (1,2). Classically, CYP11B2 was considered to utilize corticosterone generated from 11-deoxycorticosterone by action of 11β-hydroxylase (CYP11B1), but 11-deoxycorticosterone proved to be the better substrate for CYP11B2 (2).

In infancy, aldosterone synthase deficiency generally takes the form of a life-threatening electrolyte imbalance. Failure to thrive, vomiting, and severe dehydration are commonly observed in children with the condition. Two types of aldosterone synthase deficiency have been described: corticosterone methyl oxidase type 1 (CMO 1) and CMO 2. These conditions can be differentiated by the presence of insufficient or excessive 18-OH-corticosterone, respectively. CMO 1 is typically characterized by total suppression of aldosterone synthase with no detectable levels of aldosterone release. In contrast, some degree of aldosterone synthase activity persists in CMO 2 deficiency, and low to normal levels of aldosterone may be observed (3). This also explains why type 1 has a more severe course.

To the best of our knowledge, the literature contains only one case of a Turkish patient with aldosterone synthase deficiency with confirmed mutation in the CYP11B2 gene (4). In this report, we describe the second Turkish patient with aldosterone synthase deficiency who presented in infancy with failure to thrive and salt wasting. Urinary steroid characteristics were compatible with CMO I deficiency. Subsequent molecular genetic analysis on the CYP11B2 gene confirmed the diagnosis.

## CASE REPORT

Our patient was delivered normally at 38 weeks gestation with a birth weight of 3200 g and length of 50 cm. The parents are Turkish and non-consanguineous. The patient presented at the age of 2 months with vomiting and failure to thrive. On examination, his body weight was 3.800 g (3^rd^ percentile) and length was 53 cm (3^rd^ to 10^th^ percentile). She had a normal female phenotype and no hyperpigmentation was noted. Blood pressure was 80/50 mmHg.

Laboratory examination revealed a plasma sodium level of 127 mmol⁄L (136-145), plasma potassium of 5.1 mmol⁄L (3.5-5.5), and normal blood urea nitrogen and plasma creatinine. Urinary sodium levels were elevated (80 mmol/L) despite hyponatremia. Hormonal evaluation revealed cortisol of 8 µg/dL (5-25) and adrenocorticotropic hormone (ACTH) of 9.6 pg/mL (10-55). Plasma renin activity was 128 ng/mL/h (2.4-37) and plasma aldosterone 28 pg/mL (50-900). Aldosterone synthase deficiency was suspected. The patient was started on fludrocortisone (0.05 mg/day) and intravenous normal saline and responded well to treatment.

A spot urine sample was obtained before treatment, the steroid profile of which was analyzed using gas chromatography-mass spectrometry (GC-MS). This was carried out according to our previously published method (5). In brief, steroids were extracted, and conjugates were hydrolysed enzymatically using Helix pomatia digestive juice. The free steroid products were then re-extracted, and methyloxime–trimethylsilyl ether (MO–TMS) derivatives were prepared before analysis by GC-MS using a Perkin Elmer Clarus 500 system with an OV-1 column (Perkin Elmer, Beaconsfield, Buckinghamshire, UK). The steroid metabolites present in greatest quantities are quantified based on data obtained in cyclic scan mode. These comprise metabolites of androstenedione, dehydroepiandrosterone, progesterone, 17-hydroxyprogesterone, corticosterone, and cortisol.

Our patient’s urine steroid profile was typical of aldosterone synthase deficiency type 1 in that tetrahydroaldosterone, the major metabolite of aldosterone, was undetectable and corticosterone metabolites were elevated relative to cortisol metabolites, but there were no increases of 18-hydroxylated corticosterone metabolites (Table 1).

### Genetic Study

Written informed consent was obtained from the patient’s parents to participate in the study.

### Polymerase Chain Reaction and DNA Sequencing

Genomic DNA was extracted from peripheral leukocytes. All coding regions of CYP11B2 and the exon-intron splicing junction boundaries were amplified using the polymerase chain reaction method, followed by sequencing.

Sequence analysis of CYP11B2 showed that the patient was a homozygous for c.788T>A (p.Ile263Asn) and c.1157T>C (p.Val386Ala) (Figure 1A, 1B). Family genetic study showed that the mother was heterozygous for c.788T>A and homozygous for c.1157T>C, while the father was heterozygous for both c.788T>A and c.1157T>C.

## DISCUSSION

Aldosterone synthase deficiency may rarely be encountered as a cause of hyponatremia and failure to thrive in infants. When the disease is suspected, it is of vital importance that fludrocortisone therapy be initiated in addition to appropriate fluid replacement. The presence of hyperkalemia is not essential in order to establish the diagnosis. Normal potassium levels despite hyponatremia have been reported in some patients with aldosterone synthase deficiency, similar to our case (6). One recent experimental study investigated the mechanisms involved in renal control of potassium homeostasis in complete aldosterone deficiency. The results showed that renal adaptation to a physiological K (+) load in aldosterone deficiency is possible by means of aldosterone-independent activation of the renal outer medullary K (+) channel and epithelial sodium channel. Angiotensin II may also contribute to this (7).

It is difficult in infants to collect urine samples over a 24-hour period. Spot urinary steroid profiling is also useful tool to diagnose aldosterone synthase deficiency and other congenital adrenal diseases (8). Our patient’s urinary steroid profile was analyzed with a spot urinary sample, with findings consistent with CMO 1 deficiency.

Cases of aldosterone synthase deficiency have been reported in various ethnic groups, including Europeans, North Americans, and Asians (9,10,11,12). One previous case of aldosterone synthase deficiency in a patient of Turkish origin was reported from Japan (4). Ours is the first Turkish patient to be genetically confirmed and reported from Turkey. Type 1 CMO was similarly present in the other Turkish patient. We were unable to obtain detailed clinical and laboratory information from the other Turkish patient for comparison with our own case, but the mutation in the other patient was different. We identified two homozygous mutations in CYP11B2, i.e. c.1157T>C and c.788T>A. Family genetic study showed that while the father was heterozygous for these two mutations, the mother was heterozygous for c.788T>A and homozygous for c.1157T>C.

In terms of the literature concerning the homozygous mutation, p.Val386Ala (c.1157T>C), the study of Pascoe et al (13) described the variant p.Val386Ala in Iranian-Jewish kindred previously, causing a small but reproducible reduction in the production of 18-hydroxycorticosterone in vitro. They hypothesized that the presence of another mutation is required if the gene is to become defective. Therefore, despite the mother is homozygous for p.Val386Ala, the overall genetic findings in her are still consistent with her being a carrier of aldosterone synthase deficiency. Since we were unable to study our patient’s mother’s urine steroid profile, we do not know whether subtle mineralocorticoid abnormalities were present. When we inquired into her medical history, including childhood, we learned that she had no marked adrenal insufficiency, had never undergone hyponatremia attack, and had not been hospitalized. However, there is a probability of mild mineralocorticoid deficiency in the mother’s history. Another reason why the mother is today completely healthy may be that the condition gradually improves with declining renal tubular resistance to mineralocorticoids (3). Second homozygous non-synonymous variant in the patient is a novel c.788T>A (p.I263N) change which is also predicted to be disease-causing with a PolyPhen-2 score of 0.999 (http://genetics.bwh.harvard.edu/cgi-bin/pph2). This variant was neither found in ExAC nor in 1000 G.

In conclusion, this is the first report from Turkey of a Turkish patient with type 1 aldosterone synthase deficiency with a confirmed molecular basis and in which a spot urine steroid profile was used for making the diagnosis. Although aldosterone synthase deficiency is very rare, it is one of the diseases associated with hyponatremia and failure to thrive in infancy.

## Ethics

Informed Consent: Written informed consent was obtained from the patient’s parents to participate in the study.

Peer-review: Externally peer-reviewed.

## Figures and Tables

**Table 1:**
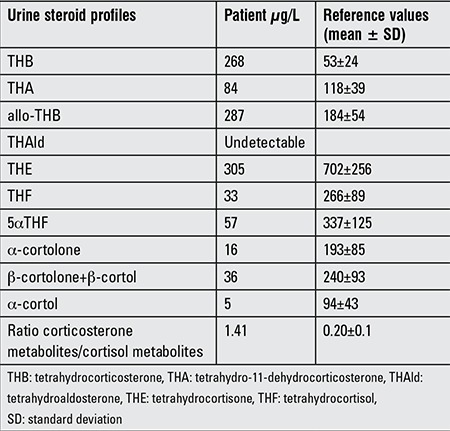
Urinary steroid profiles

**Figure 1A:**
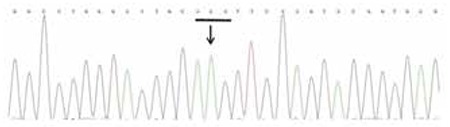
Electropherogram of segment of the CYP11B2 gene showing the mutation c.788T>A (p.Ile263Asn) in homozygous state in the patient. The affected codon is underlined

**Figure 1B:**
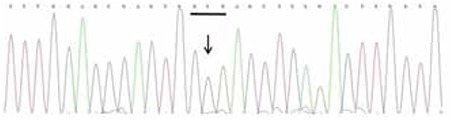
Electropherogram of segment of the CYP11B2 gene showing the mutation c.1157T>C (p.Val386Ala) in homozygous state in the patient. The affected codon is underlined
